# Exploring differences in stakeholders’ perceptions of illegal bird trapping in Cyprus

**DOI:** 10.1186/s13002-017-0194-3

**Published:** 2017-11-28

**Authors:** Heather M. Jenkins, Christos Mammides, Aidan Keane

**Affiliations:** 1 Division of Biology and Centre for Population Biology, Faculty of Life Sciences, Imperial College London, Silwood Park, Ascot, Berkshire, SL5 7PY UK; 2 Guangxi Key Laboratory of Forest Ecology and Conservation (under state evaluation status), College of Forestry, Guangxi University, Daxuedonglu 100, Nanning, 530004 China; 3 School of GeoSciences, University of Edinburgh, Edinburgh, EH9 3FF UK

**Keywords:** Birds directive, Blackcaps, Mediterranean, Migratory birds

## Abstract

**Background:**

Cyprus is recognised as a hotspot for illegal bird trapping in the Mediterranean basin. A consumer demand for the Eurasian blackcap (*Sylvia atricapilla*) is driving the use of non-selective trapping methods, resulting in the indiscriminate killing of millions of migratory birds. Efforts to tackle the issue have so far been characterised mostly by a top-down approach, focusing on legislation and enforcement. However, trapping levels are not decreasing and conflict between stakeholder groups is intensifying.

**Methods:**

To understand why efforts to stop illegal bird trapping have not been effective, we used semi-structured interviews to interview 18 local bird trappers and nine representatives from the pertinent environmental non-governmental organisations (NGOs) and the governmental agencies responsible for enforcing the legislation.

**Results:**

We found distinct differences between the views of the local trapping community and the environmental NGOs, particularly on why trapping is occurring and its impact on the avifauna. This disparity has contributed to misrepresentations of both sides and a high degree of conflict, which is potentially proving counterproductive to conservation interventions. In addition, it appears that trappers are a heterogeneous group, likely driven by various motivations besides profit.

**Conclusion:**

We argue that stakeholders interested in reducing illegal bird trapping need to develop anti-poaching strategies that aim at minimising the disparity in the views, and subsequently the conflict, acknowledging also that trappers are not a homogenous group, as often treated.

## Background

Palaearctic-African migrant birds are in decline [1–3] and evidence suggests that direct mortality from overharvesting is one of the major drivers for many of those species [1, 4, 5]. Throughout the Mediterranean, people have benefitted from the hunting of migratory birds for centuries [6], and today, both legal and illegal hunting activities result in millions of birds being shot or trapped each year as they migrate between Africa and Europe [5]. The illegal taking of wild birds is now recognised as a serious pan-European problem with clear conservation implications [7]. A range of international legal instruments and frameworks have thus been adopted to monitor and conserve wild bird populations [5], but have not yet proven successful in addressing the problem [8].

It is unclear whether this lack of success points towards the need for the current top-down enforcement strategies to be strengthened, as some stakeholders advocate [9, 10], or for a more holistic approach to be adopted—that aims to engage local communities. This dilemma largely relates to the on-going debate of how best to tackle the widespread illegal trade in wildlife [11], where increasing voices from the conservation community are advocating for the inclusion of local people in anti-poaching strategies [12, 13], as top-down enforcement strategies have similarly proved unsuccessful.

Though the issue of illegal bird trapping in the Mediterranean has not been part of this debate explicitly, it is very relevant, especially as the trapping levels continue to be high [5, 14]. A preliminary evaluation, based on data from 26 Mediterranean countries/territories, found that 11 to 36 million birds may be illegally killed or taken annually, affecting in total 456 species out of the 561 examined [5]. The reasons vary depending on the area and the species; for example, birds are illegally killed or taken for food, as a sport, for trade, and to be used as pets [5, 14, 15]. Passerine birds are impacted the most, followed by waterbirds and raptors [5].

This issue is widespread and affects almost all of the Mediterranean countries [5, 15], but is perhaps epitomised within the Famagusta District in the island of Cyprus, which has been characterised by BirdLife International [14] as “the worst in the Mediterranean area for the mean estimated number of illegally killed birds each year”. According to Brochet et al. [5], every year a mean number of 689,000 of birds are being killed illegally in the Famagusta District alone. Cyprus provides an important stopover point for an estimated 150 million migrant birds of more than 200 species, as a number of migration flyways converge over the island [16, 17]. The seasonal trapping of small migrant songbirds in Cyprus has been carried out for centuries and is therefore regarded by many within the local community as a traditional practice [16]. Once largely a fall-back food for the poor [10], blackcaps (termed locally as “ambelopoulia”) are still in high demand, providing a robust local illegal market [18]. Despite the anti-trapping legislation, which was introduced more than four decades ago (Protection and Development Game and Wild Birds Act of 1974 (No. 39/1974); [18]), recent years have seen a marked increase in trapping activities in certain areas [5], driven by the high demand for blackcaps as a traditional delicacy [15, 19].

BirdLife Cyprus, which has been carrying out systematic surveys for over 10 years [19], reports that the island’s trapping activities result in the annual, large-scale, killing of more than 2.3 million birds [14, 15]. Warblers of the genus Sylvia and particularly the Eurasian blackcap (*Sylvia atricapilia)* are targeted [15, 20], although they are not game species. The birds are trapped using limesticks and mist nets, which are illegal because of their non-selective nature. The use of this indiscriminate trapping equipment is therefore having a negative impact on numerous other species as well [15]. Although the Eurasian blackcap has a large and increasing population [21] and is therefore not classified as a threatened species, several non-target species mistakenly trapped with limesticks and mist nets are suffering from population decline and are legally protected [5, 14, 15]. BirdLife’s surveys have documented more than 152 bird species becoming caught in either type of equipment, of which 78 are listed as threatened either in the Annex I of the European Union’s Bird Directive (2009/147/EC) or in Birdlife International’s list of Species of European Conservation Concern [15].

The reported number of birds caught annually has been questioned by some of the other stakeholders, due to the multiple assumptions in the method used, particularly those associated with the practical difficulties of monitoring an illegal activity [19]. As a response, BirdLife Cyprus organised a workshop in 2015, in which foreign experts were invited to improve the method. It was concluded then that although the previously reported figures may have been overestimated, the error was unlikely to be larger than 10% [19].

Scepticism concerning the extent of the ecological impact of the trapping activities is, however, likely to persist, especially within the trapping community. Additionally, the local trappers argue that hunting Eurasian blackcaps using limesticks represents a long-held tradition, which carries for them a cultural value, and therefore they should have the right to maintain it [19]. Yet, their exact opinions and attitudes towards the issue have not been surveyed before and are largely anecdotal. To date, only a handful of scientific studies have been published on this issue, most of them more than a decade ago, aiming mainly at assessing the extent of illegal trapping [6, 16, 18], and with the social dimensions largely omitted. Little effort has been paid to understanding the multifaceted inter-relations between stakeholders, which are so often pivotal to the conservation agenda [22–24]. This study, which aims at bridging this knowledge gap, is the first to interview local people in Cyprus who are trapping birds illegally and the first to provide key insights into the motivations, attitudes and beliefs of small-scale trappers, who use the traditional trapping method, known as limesticks. It is also the first study to interview local representatives from NGOs and enforcement agencies, presenting in this way a holistic outlook of how the issue of illegal bird trapping is perceived by the majority of the key stakeholders.

## Methods

### Study area

**Fig. 1:**
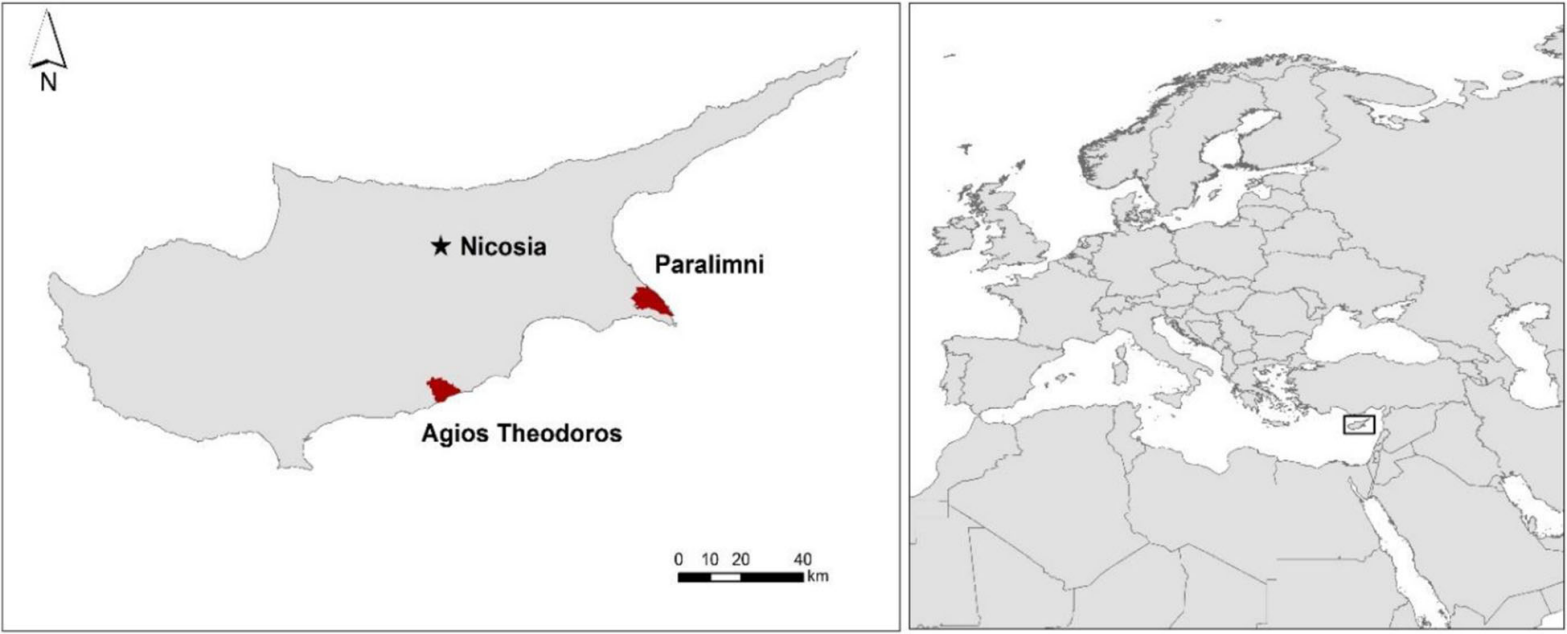
Map showing the location of the two villages in Cyprus, Agios Theodoros and Paralimni, where the interviews with the local bird trappers were conducted

Cyprus is located in the northeast corner of the Mediterranean Sea (Fig. 1), with an area of about 9250 km^2^, making it the third largest island in the Mediterranean [16, 25]. The island’s biodiversity is considered rich, as it hosts more than 1865 plant species (of which 131 are endemic) and more than 380 bird species [25]. It is part of the Mediterranean Basin biodiversity hotspot [26] and it is one of the world’s Endemic Bird Areas [25, 27]. Around 30% of the bird species of the island are known to have bred there at least once, but the majority of the birds recorded are migratory species, stopping over during their migration between Europe and Africa in the spring and the autumn [6, 16, 25, 27]. Many of these migratory species are of European and global importance and are protected under national and international legislation [10, 18].

For this study, we focused our data collection efforts on the villages of Agios Theodoros and Paralimni (Fig. 1), based on information from BirdLife Cyprus, which identified them as key trapping hotspots. Paralimni is a town located in the southeastern corner of Cyprus, within the Famagusta District, and has a total population of 14,934 (according to the population census from 2011; [28]). Of the economically active population, 13.7% is unemployed and 86.3% employed [28]. Of those employed, approximately 2% works in the primary sector (e.g., agriculture, forestry, and fishing), 15% in the secondary sector (e.g., manufacturing, and construction), and 83% in the tertiary sector (e.g., wholesale and retail trade, and accommodation and food services). The landscape in the area consists mostly of matrices of human settlements, agricultural land, and natural habitats. An Important Bird Area (IBA) and a Natura 2000 protected site, the “Paralimni Lake” [25], is located within Paralimni’s administrative boundaries.

Agios Theodoros is a village situated within the Larnaca District of Cyprus. Similarly to Paralimni, its landscape is comprised of human settlements (albeit fewer), agricultural land, and natural habitats. It also neighbours an IBA, the “Atsas-Agios Theodoros” site [17]. The village has a total population of 663. Of the economically active population, 9.3% is unemployed and 90.7% employed. Forty-three percent work in the primary sector, 19% in the secondary sector, and 38% in the tertiary sector [28]. One participant, interviewed in Agios Theodoros, lived in Mazotos, a nearby village also known for high trapping activity, with a population of 832 individuals [28]. Similarly to Paralimni, 15.9% of the economically active population is unemployed and 85.1% employed. Twenty-two percent works in the primary sector, 20% in the secondary sector, and 55% in the tertiary sector [29].

### Participants

Eighteen local trappers were interviewed in total, all of whom were Cypriot and male. Participants’ ages ranged from 31 to 90. Twelve of them worked full-time, one worked part-time, one was unemployed and the rest were retired. Ten of the participants lived in the village Paralimni, seven in Agios Theodoros and one in the village Mazotos. All of the participants reported that they trap birds on a small scale and only using limesticks, never with mist nets. Consequently, their views are only likely to be representative of the small-scale trappers who, nevertheless, based on the information collected from the NGOs and the enforcement agencies, most likely represent the majority of the trappers, although not necessarily the trappers with the largest impact (Table 1).

**Table 1:** Summary of ‘trapper categories’, as defined by the key informants of the enforcement agencies and the environmental NGOs, indicating (a) an estimate of the number of people involved, (b) the possible motives, (c) estimates of the equipment they use and (d) the impact they may have

	Categories	Number of people trapping		Incentives	Equipment (per person)	Numbers of birds trapped/impact
Enforcement Agency 1	1) Traditional trappers	–		Personal consumption	20–30 limesticks	The large number of low-scale trapping has a significant impact
	2) Organised criminals	40–50 people in total (4–5%)		Profit	Maybe 30 mist nets and 500 limesticks	Highest impact, as catching most amount of birds
Enforcement Agency 2	1) Non-professionals	–		Personal consumption	–	–
	2) Professional trappers	10–15 people in total (within the SBA)		Profit	–	–
Enforcement Agency 3	1) Traditional trappers	A lot more than 2000 people in total		Personal consumption/hobby	Limesticks	Catch a limited number of birds as they do not use lures
	2) Business-scale			Profit	–	–
Environmental NGO 1	1) Small-scale trappers	60–85%	500 to 1000 in total	Hobby	< 50 limesticks and/or 1 mist net	c. 2 million birds in total
	2) Medium-scale trappers	10–30%		Supplementary income	50–100 limesticks and/or 1–3 mist nets	
	3) Big trappers (professionals)	5–10% (10–20 people)		Profit	>100 limesticks and/or 4+ mist nets	
Environmental NGO 2	1) Small-scale trappers	50–60%	1500 to 2000 in total	Personal consumption	25–50 limesticks or 1 mist net and 1 decoy	c. 2 million birds in total
	2) Medium/semi-professional trappers	30–40%		Personal consumption/ profit Political rather than cultural incentive	75–100 limesticks, 2–3 mist nets and 2 decoys	
	3) Professional trappers	10–20%		Profit	200 limesticks, more than 5 mist nets	

The information provided in this table reflects the opinions, knowledge and experience of the different stakeholders. Please note that two of the environmental NGOs did not have relevant information to provide

‘*–*’ = no information was provided

Although the main aim of this study was to interview the local trappers, to understand better their motivations and attitudes towards illegal bird trapping, we additionally interviewed nine key informants from four non-governmental environmental organisations (NGOs) involved in the campaigns against illegal-bird trapping, and three governmental agencies, responsible for enforcing the legislation. We did this to obtain a more balanced perspective on the issue of illegal bird trapping in Cyprus and to understand better the differences in stakeholders’ perceptions. Those organisations were BirdLife Cyprus, the Committee Against Bird Slaughter (CABS), Friends of the Earth, Terra Cypria, Game Fund, the Anti-Poaching Police Unit of the Republic of Cyprus, and the British Sovereign Base Areas (SBA) Police Service.

### Data collection and analysis

For all the interviews, we chose to use semi-structured interviews to enable the exploration of individual motivations and attitudes towards various issues surrounding illegal trapping. This method allowed for themes and topics to emerge whilst enabling the informants to express their thoughts and opinions by answering from their own frame of reference [30]. Semi-structured interviews are valuable when investigating sensitive topics and are considered less threatening than questionnaires [31]. They offer the opportunity for participants to talk freely, thus enabling the researchers to gather background information and context while collecting in-depth information on each participant’s views, perspectives and motivations [32]. All interviews were conducted by HJ and CM, a native Greek speaker, between May 29 and June 26, 2013. The interviews with local trappers were conducted in Greek as most did not speak English. HJ first asked the question in English and CM repeated it in Greek. The local trapper’s response was then translated to English by CM, allowing written notes to be taken by HJ while the interview was conducted. The interviews with key informants from the NGOs and the governmental agencies were conducted in English by HJ, in the presence of CM. The interviews in English were recorded and transcribed later by HJ. In order to facilitate discussion, individual question guides were used to ensure the main points were covered.

Purposive sampling was used, whereby participants with specific characteristics relevant to the study were intentionally selected, as they were likely to be most informative [32, 33]. In particular, selected participants had to be involved in past or present trapping activities and reside in communities with strong trapping culture. Respondent-driven sampling was also appropriate given the sensitive nature of the research and subsequent small sample size. Local trappers were invited to participate via a single informant, a well-respected trapper, who made initial contact and encouraged other people to take part. These individuals subsequently let others know of the study and encouraged them to respond to the interview request. Participants were contacted based on whether they were currently or had ever partaken in the trapping of blackcaps. It was important to speak to people directly involved in managing the issue of illegal trapping. Key informants from NGOs and enforcement agencies were therefore contacted directly and meetings arranged over the telephone.

The data was analysed by HJ using the software analysis tool NVivo 10, which enabled the organisation of complex data (collected from both interviews with the local trappers and key informants) into emerging themes by means of coding. Coding was performed by HJ and used to identify patterns or themes within the data through highlighting normative statements, interesting facts and areas of disagreement [32]. Codes were categorised hierarchically with a small number of top-level codes representing the key themes, a group of subcategories according to source and, finally, different attitudes on each particular theme/topic.

## Results

### ‘Trapper categories’ and feelings of misrepresentation

In contrast to most of the current campaigns against bird trapping, which do not appear to distinguish between the groups within the trapping community, key informants from the governmental and non-governmental bodies describe a range of ‘trapper categories’, from small-scale to professional (Table 1). Each category is intended to loosely represent a subgroup of trappers who share similar characteristics, such as incentives, type and amount of equipment used and the subsequent number of birds trapped (Table 1). Every trapper interviewed expressed the opinion that environmental NGOs and the media often misrepresent the trapping community by exaggerating the number of birds being trapped, portraying them all as being engaged in large-scale trapping activities, driven only by profit, and dismissing other non-monetary motivations. They felt that it was the more extreme types of trapping activity being presented, such as the use of mist nets, which they felt is not representative of the trapping community. ‘They never portray the situation correctly. If they want to say something about the topic they usually show mist nets and that is not always the reality’ (Trapper 3, age 53).

### Motivations for trapping

**Fig. 2:**
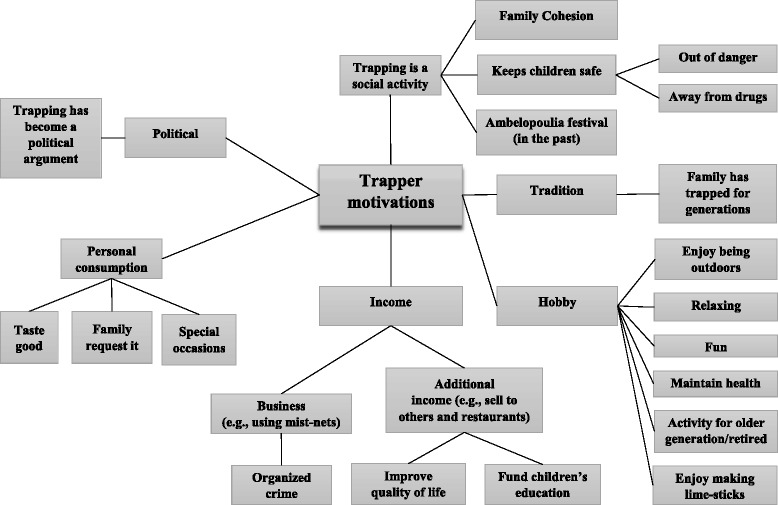
Diagram summarising the range of potential motivations for trapping birds, as described by all stakeholder groups in the study

Within the two villages sampled (Agios Theodoros and Paralimni), all 18 interviewees described a strong history and tradition going back many generations of people trapping and consuming blackcaps, using limesticks. Besides trapping birds for personal consumption, the activity has also been a significant source of income, supporting local livelihoods and in recent years, funding their children’s higher education. As one trapper mentioned: ‘People make a profit out of selling birds. This is a family town and the money is saved to have the kids educated, to improve their lives (Trapper 7, age 55). Other motivations for trapping blackcaps also exist, which are not, however, solely income related (Fig. 2). For instance, three trappers mentioned that they enjoy trapping, referring to it as a hobby that they do to relax and claimed that they only catch a small number of birds. ‘For me, personally it is a way to maintain my health. When I go out I put out 10–15 limesticks, I forget about everything else and I relax’ (Trapper 10, age 55). The process of making the limesticks, preparing the orchard, trapping and then consuming ambelopoulia was described as being an important social activity that most could remember doing with their fathers and grandfathers since a very young age. Another trapper mentioned: ‘I remember when I was a kid the whole extended family would prepare the limesticks. It was a very nice occasion for family gatherings and helped keep the family together. It was important for family cohesion’ (Trapper 13, age 31). The NGOs’ key informants on the other hand, argued that trappers’ main motivations for catching birds are for personal consumption and profit (Table 1); other motivations were not mentioned as important.

### Trappers’ knowledge of the law

The interviews with the trappers revealed gaps in knowledge regarding the laws regulating trapping activities and the potential subsequent ecological impact that trapping can have on the populations of vulnerable species [5]. The local trappers expressed a range of opinions in response to why they think that trapping is illegal (Table 2). Five participants responded that Cyprus had to prohibit the trapping of blackcaps due to pressure from the European Union and because they did not apply for a derogation during the accession. The second most common response was that people just did not know. None of the trappers referred to the indiscriminate nature of the trapping equipment as a reason why trapping is listed as illegal under the national law and the Birds Directive (Directive 2009/147/EC; [10]).

**Table 2:** A selection of trapper responses as to why they think that trapping is illegal (in order of frequency)

Number of times the response was given	‘Why do you think that it is illegal to trap birds?’
5	*I think that it is because we agreed upon a EU directive without asking for a derogation from the law to trap on a traditional basis*
3	*I don’t know*
2	*The only reason it is illegal is because they haven’t found a way to tax it yet and because they assume that some of us are making a large amount of money out of this, which is not true*
2	*I am very aware of the laws. It happened during the Bern Convention when all states decided to protect birds that are less than 7.5 cm (including ambelopoulia)*
1	*The government had to prohibit the trapping and consumption of ambelopoulia because of pressure from the EU and threats that their tourism will be affected*
1	*English used to live here and they made the law 50–200 years ago and it still runs today and goes on and on*
1	*It’s all about the money. They decided to prohibit it because they thought the people were making too much money out of it. I don’t think that it is about protecting the birds because I don’t think that there is a risk to the birds. I think that it is about the high prices*
1	*It’s because of the media’s exaggeration and misinformation*
1	*It has always been illegal but people were allowed to do it in the past*

### Trappers’ knowledge of the ecological impacts

When discussing whether traditional trapping practices, using limesticks, have an impact on bird populations, 13 of the trappers interviewed responded quite emphatically that this was not the case and described the long tradition as evidence for this. ‘It has been proven that people in Cyprus have been capturing birds using these traditional methods since the 16th century so if there was an impact then we wouldn’t see this many birds around’ (Trapper 10, age 55). Twelve trappers openly condemned the use of non-traditional methods, particularly mist nets, acknowledging their potential for negatively impacting birds. ‘It is right to be concerned because of the mist nets and decoys, but the limesticks do not have an effect, as it is something that has been happening for thousands of years now’ (Trapper 1, age 58).

Although the non-selective nature of the equipment was not identified by any of the trappers interviewed as a potential problem, every NGO representative interviewed emphasised the non-selective nature of all the trapping equipment used as what makes the activity potentially so damaging—since it can reduce populations of vulnerable species, result in the local extirpation of species, and result in the loss of genetic diversity [5]. Trappers’ responses concerning the actual number of other bird species caught for every blackcap displayed considerable variation, but 11 of the trappers interviewed stated that trapping species other than blackcaps was not a frequent occurrence. ‘It’s rare to catch other species and because of the nature of the limestick you cannot catch any big birds. If we sometimes catch a bird that is not a blackcap and it is not suitable for consumption we release it’ (Trapper 9, Age 47). NGO representatives disagreed with this claim because field surveys have shown that both limesticks and mist nets often capture a wide range of species, not just birds but also reptiles [15]. Moreover, some of the NGO representatives maintained that because freeing birds captured on limesticks is a particularly challenging and time-consuming task, it is highly unlikely that trappers release any birds when they realize that those caught do not belong to the targeted species.

### Stakeholders’ views on the law and enforcement measures

The majority of local trappers interviewed (14 out of 18) considered the current laws regarding trapping practices to be ‘unfair’ with almost everybody saying that the fines were too high. ‘The laws are very, very strict especially for low use of limesticks. It is unacceptable to catch somebody with 10 birds and fine them for €3,000’ (Trapper 3, age 53)*.* Comparisons were frequently made between trapping and other illegal activities, such as drug use. They often gave anecdotal evidence about people who were caught dealing or using drugs but given a comparatively smaller fine. ‘For example, this happened to me personally, they caught me with one bundle of limesticks [24] and 4–5 birds and they fined me €1200 euros while at the same time they gave a €600 fine to somebody who was dealing drugs’ (Trapper 14, age 54).

Contrastingly, the NGOs and enforcement agencies believe that the fines are not high enough and described the weak judicial system as a major challenge to effective enforcement. The final stage of enforcement involves the court procedure and any person accused of illegal trapping for the first time faces a potential fine of up to **€**17,000 and 3 years in jail [34]. The enforcement agencies and environmental organisations described the reality quite differently. One member of the Anti-Poaching Unit (APU), possibly referring to large-scale trappers, described their frustration with the situation: ‘It is very easy to find a loophole in the law. We follow the procedure and they go to court, but they manage to escape paying a big fine, instead only paying a small amount in comparison to how much money they are making. It is not a big deal for them to pay **€**4,000 or something similar’. Although it is difficult to know how much money individual trappers make by catching and selling blackcaps, the total worth of this illegal activity has been estimated by the authorities to be around 15 million euros annually [10, 34]. It is not known however what percentage of that goes to large-scale trappers, who use mostly mist nets and decoys to lure birds, and what percentage goes to trappers who catch birds at a smaller scale using limesticks.

Most NGOs and enforcement agencies also identified the length of time between arrest and court trials as a significant factor leading to non-deterrent prosecutions. The APU stated the following: ‘You arrest somebody and it might take two years for them to face trial, during which time they continue making an income from trapping. You might catch them another four times during this period and it looks like he has been caught once as the court just puts them all together’.

### Contentious conservation

The role of conservation organisations in the trapping debate is highly contentious in Cyprus, particularly within the village of Agios Theodoros and villages in Famagusta District. According to the trappers interviewed, local people are not supportive of the conservation efforts carried out by organisations such as CABS, and there is a great deal of tension between the two stakeholder groups, sometimes even resulting in physical confrontations as reported multiple times in the local media [20, 35]. Local trappers often expressed scepticism when asked about their attitudes towards the motivations of the environmentalists, suggesting in order of frequency that (1) they have a financial incentive to do this work; (2) their aim is to create a negative image of the people trapping; (3) ‘they have nothing better to do’ and finally (4) they do it to preserve the birds. On the other end of the spectrum, based on our interviews with the key informants from the environmental NGOs, conservationists seem to have the opinion that the non-monetary motives for trapping, expressed by the locals, are minor and unimportant. Most advocate for zero tolerance and stricter law enforcement [10], and treat local trappers as a homogenous group, driven by the same motives, mostly conducting an illegal activity on a large scale merely for profit.

## Discussion

It is evident from the responses of the local trappers, the representatives of the environmental NGOs and the management agencies, that the human dimensions of the issue of illegal bird trapping in Cyprus are complex and conflicting. Our work describes the beliefs and attitudes of the groups involved, and allows us to understand the dynamics that are ultimately shaping the way in which stakeholders are behaving and reacting to this important conservation issue.

### Lack of understanding and trust

It is reasonable to suggest, since trapping levels are still high [15], that current anti-poaching measures are not proving successful. Although several factors could be contributing to this lack of success, such as the absence of strong will on behalf of politicians [19, 38] and insufficient law enforcement resources [36–38], we believe that the lack of understanding and trust between the trapping community and the conservationists is a key stumbling block inhibiting conservation success [39]. The communication gap between the two groups acts as a breeding ground for high stakeholder conflict [22] and allows for misunderstandings on the issue to persist, such us on why the practise is illegal and what the potential ecological impact is, especially on threatened species. This is illustrated by no trapper suggesting the indiscriminate nature of limesticks as a reason for their being illegal, despite the fact that this is a key part of the reasoning behind the prohibition of limesticks under national and international law (Protection and Development Game and Wild Birds Act of 1974 (No. 39/1974); [18]). The non-selective nature of the trapping activities is one of the two key messages that NGO’s aim to communicate, the second being the large scale at which trapping is occurring [15]. Although it is possible that members of the trapping community are choosing to ignore or not understand this aspect (as it is against their interests to do so), it is probable that the lack of trust and communication between the two groups, which is exacerbated by misrepresentations, is preventing the message from reaching the community.

### Imprecise portrayal of the trappers

The interviewed tappers felt strongly that their portrayal in the anti-poaching campaigns and the media is unfair and unrepresentative, i.e., as organised criminals trapping birds on a large scale and being driven only by profit. Although such groups do exist, according to most of the stakeholder groups interviewed, those that engage in ‘professional’ or ‘large-scale’ trapping for profit, constitute between 5 and 20% of the total trapping community (Table 1). It is however, this image that the environmental NGOs and the media portray, making it appear to the public as the primary form and reason for trapping. The presence of such organised trapping activities and its impact on bird populations is likely to be significant [5, 15] and it requires different anti-trapping strategies than the rest of the trapping community. Using the same approach for all trappers and treating them as one homogenous group with the same motive is neither accurate nor effective.

Each participant of this study described his own connection with trapping, explaining its importance at the personal and also often at the village level within a strong historical context. It is clear that this activity is often highly valued for both its intrinsic sociocultural and economic value (Fig. 2). For the development of effective conservation measures, which should be tailored to each trapping subgroup, it is necessary that these values are understood and not discounted [8, 40]. The failure of most anti-trapping campaigns to accurately present and account for the different categories of trappers and their diverse motives, has possibly created a credibility gap for the conservation advocates. This loss of credibility, in addition to the conflict, may have resulted in the trappers and potentially the general public dismissing the campaigners’ conservation messages, making addressing the problem even more challenging.

Another apparent challenge is the lack of key data, essential for understanding better the issue and the characteristics of each trapper subgroup. Currently, it is still unclear what the actual number of trappers is, what percentage of those trap birds on a small scale, for example for personal consumption only, and what percentage trap birds on a larger scale for illegal trade and profit. It is also unclear how many trappers use limesticks vs. mist nets, how often, what percentage of birds are trapped with each method and what percentage in each case is traded.

## Conclusions

The conservation community is increasingly recognising that issues such as poaching and wildlife trade are multifaceted [29] and driven by complex social, cultural and economic factors [39, 41, 42]. Overreliance on enforcement measures not only fails to address these complexities, but also can prove counterproductive by, for example, driving trade further ‘underground’ [41]. Although we acknowledge that environmental NGOs are correct in identifying lack of political will [10] and insufficient enforcement of the current laws as factors hindering conservation success [43], we argue that anti-trapping efforts need to be adjusted and acknowledge the realities on the ground and the differences between the trappers. Efforts must account for the complex social dimensions [8, 24] behind this conservation issue and engagement of the local communities is needed where trapping occurs the most. It is therefore suggested to adopt a more inclusive, participatory approach that aims to recognise the views of stakeholders at local, national, and global levels. Efforts should simultaneously be made to better address the drivers of poaching and empower local communities, through innovative and alternative schemes, to participate in the protection or sustainable management of wildlife populations. Given the transboundary nature of Palearctic-African migrant birds, such strategies will prove most effective when undertaken across their entire range [1]. Thus, the use of a holistic approach and recognising the importance of understanding these underlying human dimensions should also be applied within its widest possible context.

## Abbreviations

APU: Anti-Poaching Unit
CABS: Committee Against Bird Slaughter
IBA: Important Bird Area
NGOs: Non-governmental organisations
SBA: Sovereign Base Areas
